# The Steric Gate of DNA Polymerase ι Regulates Ribonucleotide Incorporation and Deoxyribonucleotide Fidelity*

**DOI:** 10.1074/jbc.M113.545442

**Published:** 2014-02-14

**Authors:** Katherine A. Donigan, Mary P. McLenigan, Wei Yang, Myron F. Goodman, Roger Woodgate

**Affiliations:** From the ‡ Laboratory of Genomic Integrity, NICHD and; the § Laboratory of Molecular Biology, NICHD, National Institutes of Health, Bethesda, Maryland 20892 and; the ¶ Department of Biological Sciences and Department of Chemistry, University of Southern California, University Park, Los Angeles, California 90089

**Keywords:** DNA-binding Protein, DNA Repair, DNA Synthesis, Enzyme Kinetics, Mutagenesis, DNA Polymerase Iota, Ribonucleotide Incorporation, Steric Gate Mutant, Y Family DNA Polymerase

## Abstract

Accurate DNA synthesis *in vivo* depends on the ability of DNA polymerases to select dNTPs from a nucleotide pool dominated by NTPs. High fidelity replicative polymerases have evolved to efficiently exclude NTPs while copying long stretches of undamaged DNA. However, to bypass DNA damage, cells utilize specialized low fidelity polymerases to perform translesion DNA synthesis (TLS). Of interest is human DNA polymerase ι (pol ι), which has been implicated in TLS of oxidative and UV-induced lesions. Here, we evaluate the ability of pol ι to incorporate NTPs during DNA synthesis. pol ι incorporates and extends NTPs opposite damaged and undamaged template bases in a template-specific manner. The Y39A “steric gate” pol ι mutant is considerably more active in the presence of Mn^2+^ compared with Mg^2+^ and exhibits a marked increase in NTP incorporation and extension, and surprisingly, it also exhibits increased dNTP base selectivity. Our results indicate that a single residue in pol ι is able to discriminate between NTPs and dNTPs during DNA synthesis. Because wild-type pol ι incorporates NTPs in a template-specific manner, certain DNA sequences may be “at risk” for elevated mutagenesis during pol ι-dependent TLS. Molecular modeling indicates that the constricted active site of wild-type pol ι becomes more spacious in the Y39A variant. Therefore, the Y39A substitution not only permits incorporation of ribonucleotides but also causes the enzyme to favor faithful Watson-Crick base pairing over mutagenic configurations.

## Teaser

**Background:** Accurate bypass of DNA damage by translesion DNA polymerases is critical for cell survival.

**Results:** Wild-type human DNA polymerase ι incorporates ribonucleotides, and a steric gate mutant increases both ribonucleotide incorporation and deoxyribonucleotide selectivity.

**Conclusion:** A single amino acid residue in DNA polymerase ι limits incorporation of ribonucleotides into DNA.

**Significance:** DNA polymerase ι may incorporate ribonucleotides during translesion DNA synthesis.

## Introduction

The daily rate of DNA damage is estimated at upwards of 50,000 lesions per cell (1). Although DNA repair mechanisms evolved to contend with this high rate of damage, lesions that persist may stall replication, leading to fork collapse, double-strand breaks, and genomic instability. Such instability is a hallmark of tumorigenesis. To prevent stalling at sites of damage, cells utilize specialized DNA polymerases (pols)^2^ to promote translesion synthesis (TLS) that enables bypass of damage and prevents genomic instability (2). During DNA replication, pausing by the replicase leads to a polymerase switch such that the replicase is replaced by a TLS polymerase capable of bypassing the damage (3). After the damage has been bypassed, the distributive TLS polymerase may synthesize only a handful of nucleotides before dissociating from the DNA, thereby allowing the replicase to resume genome duplication. This mechanism of DNA damage tolerance is critical to maintaining genomic integrity and, ultimately, cell survival.

Many TLS specific polymerases are characterized by lower fidelity and processivity on undamaged DNA than replicases. One of the most error-prone human DNA polymerases is the Y family enzyme, DNA polymerase ι (pol ι). The distributive enzyme lacks exonuclease activity and has a distinct misincorporation signature that is template-dependent (4). For example, misinsertion of G opposite template T occurs with 3–10-fold greater efficiency than insertion of the correct A nucleotide, which causes the enzyme to abort or pause synthesis at template T (5). In contrast, pol ι exhibits its most efficient correct incorporation opposite template A (5). Unlike other Y family members, pol ι also possesses intrinsic dRP-lyase activity and as such may participate in specialized forms of base excision repair *in vivo*, potentially during the somatic hypermutation process (6, 7). pol ι has also been implicated in the TLS of oxidative and ultraviolet-induced DNA lesions (8–11). Crystal structures of the catalytic domain of pol ι show that the unique active site of the enzyme readily facilitates Hoogsteen base pairing to accommodate DNA lesions and mispairs (12–14). pol ι has been shown to prefer manganese over magnesium as the divalent cation for catalysis and is highly active with low levels (50–250 μm) of manganese (15, 16).

Genomic stability depends on DNA polymerases selecting the correct dNTP for the template and the exclusion of NTPs, because cellular NTP levels are much higher than dNTPs (17, 18). This imbalance necessitates the critical ability of DNA polymerases to prevent incorporation of NTPs, especially because they are highly susceptible to spontaneous hydrolysis and render the DNA at risk for breaks. Surprisingly, NTPs appear to be tolerated to an extent in DNA, because ribonucleotides have recently been shown to be the most common “lesion” in the mouse genome (19). Studies in yeast on high fidelity replicative polymerases α, δ, and ϵ estimate that these enzymes incorporate one NTP for every 625–5,000 dNTPs, resulting in over 10,000 NTPs incorporated into the genome during one round of replication (20, 21). Human pol β, a comparatively lower fidelity enzyme involved in base excision repair, has been reported to insert one NTP per 81 insertion events (22). Removal of ribonucleotides from DNA is accomplished by RNase enzymes in a pathway of ribonucleotide excision repair (23).

Many DNA polymerases contain a single residue nascent to the active site that functions as a “steric gate,” thereby preventing incorporation of NTPs by steric hindrance with the 2′-OH that does not exist with dNTPs. The location of this residue is highly conserved within polymerase families (24). *Escherichia coli* pol V is a highly error-prone, TLS polymerase that is homologous to Y family polymerases in eukaryotes. Our laboratory has previously identified critical amino acids in pol V that control sugar discrimination (25). Surprisingly, we found that even the wild-type pol V enzyme efficiently incorporates NTPs into DNA. A single conserved aromatic residue, Tyr^11^, which was identified as the steric gate (26), was shown to limit NTP incorporation into DNA, because substitution with the smaller alanine amino acid further increased NTP incorporation by pol V. In contrast, when the adjacent highly conserved Phe^10^ residue was changed to leucine, it had the opposite effect and restricted NTP incorporation and also increased dNTP selectivity.

**FIGURE 1.**
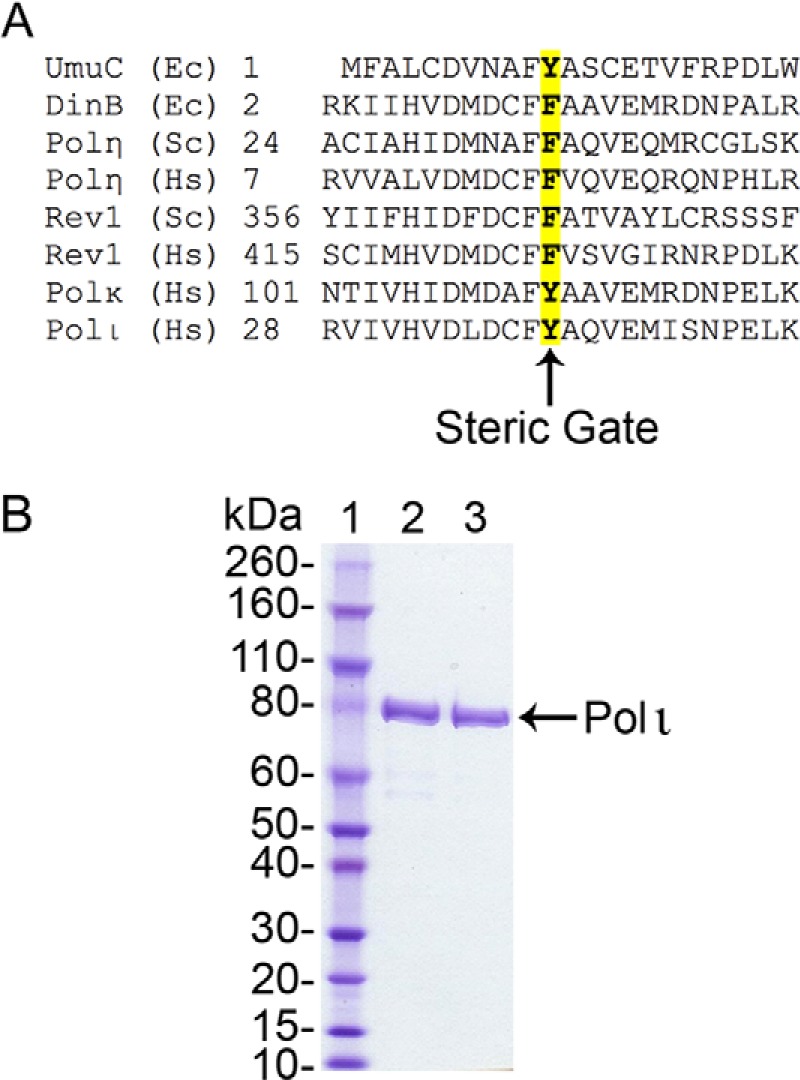
*A*, sequence alignment of the conserved steric gate residues of Y family TLS polymerases. *B*, SDS-PAGE analysis of final enzyme preparations. 3 μg of wild-type pol ι (*lane 2*) and the pol ι Y39A variant (*lane 3*) are shown.

In this work, we investigated the NTP insertion efficiency and fidelity of the human Y family TLS enzyme, pol ι. We further examined the structural basis for NTP exclusion using a steric gate mutant, that based upon residues conserved in Y family DNA polymerases is identified as Tyr^39^ in pol ι (Fig. 1*A*). Wild-type pol ι and the Y39A variant were evaluated for their ability to bind DNA and both incorporate and extend NTPs *in vitro* using replication assays, steady-state kinetics, and gel mobility shift assays. Like *E. coli* pol V, we found that human pol ι readily incorporates ribonucleotides into DNA. As expected, ribonucleotide incorporation increased in the Y39A variant. Surprisingly, dNTP selectivity also improved in the Y39A mutant. Molecular modeling suggests that when compared with the constricted active site of the wild-type enzyme, which normally favors highly mutagenic base pairing, the Y39A active site is more spacious, thereby allowing normal and accurate Watson-Crick base pairing to occur.

## EXPERIMENTAL PROCEDURES

### 

#### 

##### Materials

Ultrapure NTPs and dNTPs were purchased from GE Healthcare and Roche, respectively. All oligonucleotides were synthesized by Lofstrand Laboratories (Gaithersburg, MD) and gel-purified prior to use.

##### DNA Substrate Preparation

Primers were 5′-radiolabeled with ^32^P by Lofstrand Laboratories. Annealing reactions consisted of 100 nm radiolabeled primer and 200 nm unlabeled template in 50 mm Tris-HCl, pH 8.0, 5 mm NaCl, 50 μg/ml bovine serum albumin, and 1.42 mm 2-mercaptoethanol. Reaction mixtures were heated at 95 °C for 5 min and then slowly cooled to room temperature over several hours.

##### Protein Expression and Purification

Full-length N-terminal His-tagged human wild-type pol ι was expressed using the *E. coli* strain RW644 and expression plasmid pJM868 as previously described (27). The Y39A pol ι mutant was generated by chemically synthesizing a 310-bp NcoI-BglII fragment encoding the N terminus of the *E. coli* codon optimized *POLI* gene with the Y39A substitution (Genscript, Piscataway, NJ). The fragment was subsequently subcloned as an NcoI-BglII fragment into pCT14 (a precursor of pJM868 with an NcoI site at the start of the *POLI* gene), and the resulting plasmid, pKD001, was introduced into the expression strain RW644, and the Y39A pol ι was protein-purified using the same protocol as wild-type pol ι (27). Final preparations of both wild-type pol ι and Y39A were determined to be >95% pure by SDS-PAGE gel analysis (Fig. 1*B*). Protein concentrations were determined using the Bio-Rad protein assay designed for 96-well plates and a plate reader. Commercially prepared His-tagged full-length pol ι (ProteinOne, Gaithersburg, MD) was used to generate the standard curve. Final preparations for both wild-type pol ι and Y39A were determined to be 1–2 μm.

##### Primer Extension Reactions

Standard reaction mixtures (10 μl) contained 100 nm pol ι, 10 nm of radiolabeled DNA substrate, 100 μm nucleotide(s) in 1× reaction buffer (0.25 mm MnCl_2_, 40 mm Tris-HCl, pH 8.0, 10 mm dithiothreitol, 250 μg/ml bovine serum albumin, 2.5% glycerol). All DNA substrates were confirmed to be >95% annealed by incubating with wild-type pol ι and 100 μm of all four dNTPs. The reactions were incubated at 37 °C for 10 min and quenched by the addition of 10 μl of 95% formamide, 10 mm EDTA solution. The samples were heated to 100 °C for 5 min and resolved on an 18% polyacrylamide, 8 m urea gel. Reaction products were visualized and quantified using a Fuji FLA-5100 Phosphorimager and ImageGauge software.

##### Steady-state Kinetic Assays

Incorporation of select dNTPs or NTPs opposite template G and T was evaluated using steady-state kinetics. Steady-state kinetic parameters *k*_cat_ and *K_m_* were measured using standing start reactions as previously described (28). All reactions contained 10 nm DNA substrate and 1× reaction buffer. Reaction conditions were optimized using a time course for a variety of enzyme:DNA ratios to ensure that reactions were in the linear range (<20% of total primer extension). Steady-state reactions measuring correct dNTP incorporation were performed for 1–2 min using 0.4–1 nm enzyme. Reactions examining incorrect dNTP incorporation were performed for 1–7.5 min using 1.5–10 nm enzyme. Reactions examining NTP incorporation were performed for 1–20 min using 1–10 nm enzyme. Nucleotide concentrations included in reactions ranged from 0.001 to 100 μm. All reactions were initiated by mixing equal volumes of 2× enzyme/DNA solution with 2× nucleotide/Mn^2+^ solution. All 2× solutions were preincubated at 37 °C prior to the reaction. The reactions were quenched, visualized, and quantified as described above. The data were plotted using GraphPad Prism (v6.0) software (San Diego, CA). By plotting velocity (*v*) *versus* dNTP concentration and fitting the Michaelis-Menten rectangular hyperbola by least squares (29), we obtain the kinetic parameters *V*_max_ and *K_m_*.

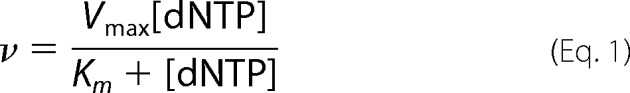
 The misincorporation frequency for an incorrect dNTP is calculated as follows,

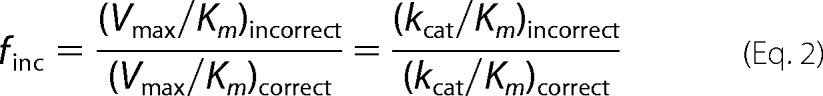
 where *V*_max_ = *k*_cat_ [total polymerase-primer-template complex] (29). Each reported value is the average of at least three independent experiments.

##### Electrophoretic Mobility Shift Assays

DNA binding constants (*K_D_*_(DNA)_) for wild type and Y39A were determined by gel electrophoretic mobility shift assay as described (30) with several modifications. Radiolabeled DNA substrate (0.2 nm) was incubated with a dilution series of pol ι enzyme (0.2–780 nm) in 1× reaction buffer for 20 min at 25 °C. Samples were loaded onto a 6% Native Tris acetate PAGE gel that had been prerun at 150 V at 4 °C for 1 h. The gels were run at 150V for 4–5 h at 4 °C, then dried, and exposed for 2 h before phosphorimaging. The fraction of bound DNA relative to total DNA was quantified using ImageQuant software. The fraction of bound DNA was plotted as a function of protein concentration, and the DNA binding constant was determined by fitting data to the following equation,

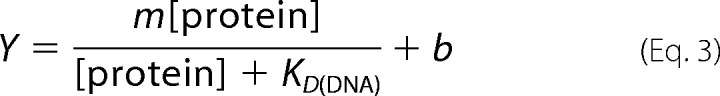
 where *Y* is the amount of bound protein, *m* is a scaling factor, and *b* is the apparent minimum *Y* value (31).

##### Replication Foci Assay

Recruitment of Y39A pol ι to nuclear replication foci following UV treatment was assessed as previously described (32). The peCFP-C1-pol ι construct encoding the CFP-ι fusion protein has been previously described (33). To obtain a construct encoding the CFP-ι fusion protein with the Y39A substitution, a 204-bp BglII-BstEII fragment with the desired sequence was chemically synthesized (Genscript) and subcloned into the wild-type peCFP-C1-pol ι vector to generate pKD004. Constructs were transfected into MRC5 fibroblasts using TurboFectin 8.0 according to the manufacturer's instructions (Origene, Rockville, MD). For UV treatment, cells were irradiated 20 h post-transfection at 7 J/m^2^ and recovered by incubating for a further 6 h. Cells were fixed as noted (33). Localization of pol ι, as indicated by CFP fluorescence, was evaluated by fluorescence microscopy using a Zeiss Axiophot2 microscope (Carl Zeiss), an Orca ER CCD camera, and Simple PCI software (Hamamatsu). Slides were imaged by excitation at 436 nm and CFP emission at 480 nm. At least 200 nuclei were counted for each cell line and treatment from three independent experiments.

##### Molecular Modeling

The structure of pol ι in a ternary complex with a template dG and incoming dCTP has been determined (PDB code 2ALZ) and requires no modeling (14). The structure of pol ι in a ternary complex with a template dT and incoming dATP was modeled on the BrU/dGTP (PDB code 3H4D) published structures (34). The BrU is replaced by a normal thymine, and the dGTP is replaced by dATP. The misincorporation of dGTP opposite dT was based on the ternary complex structure of pol ι with a template dT and incoming dGTP (PDB code 3GV8) and requires no remodeling (12). The misincorporation of dGTP opposite dG was based on the ternary complex structure of pol ι with a template 8-oxo-G and incoming dGTP (PDB code 3Q8R) and incoming dATP (PDB code 3Q8Q), which shows more deviation of the deoxyribose from the standard conformation (8). The 8-oxo-G was converted to a normal G. All four structures were superimposed, and the protein structures are highly similar. Because of the narrow active site for the replicating base pair, large deviations are found in the positions of incoming dNTP. In the majority of the structures, the α, β, and γ triphosphate superimposed well. Deviations occur most often in the deoxyribose moiety (8), as exemplified in Fig. 9*E* when G:dGTP or A:dGTP mismatch occurs. All ribonucleotides are modeled based on deoxyribonucleotide by replacement of a deoxyribose by a ribose.

## RESULTS

### 

#### 

##### Residue Tyr^39^ Functions as a Steric Gate to Impede Ribonucleotide Incorporation and Its Reduced Catalytic Activity Is Rescued by Mn^2+^

**FIGURE 2.**
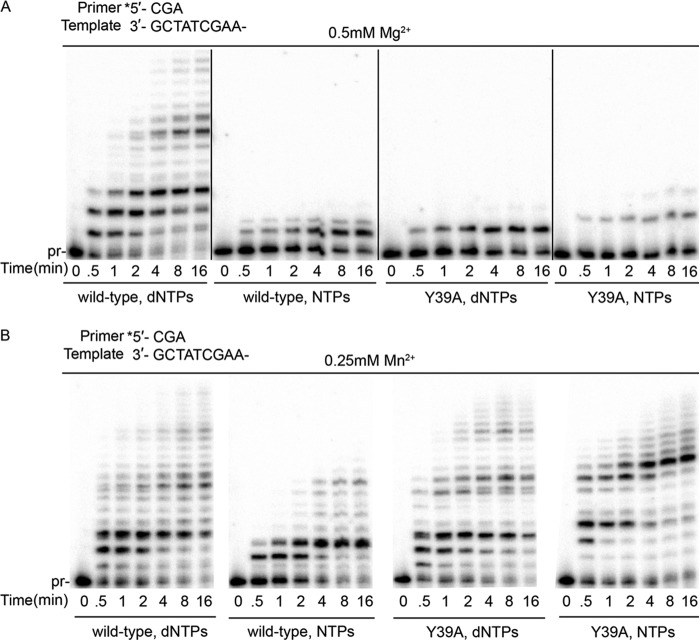
**Mn^2+^ can rescue the catalytic activity of the pol ι Y39A mutant.** Primer extension assays in the presence of Mg^2+^ (*A*) or Mn^2+^ (*B*) were performed with the enzymes, nucleotides, and DNA substrates indicated.

To assess the ability of full-length wild-type pol ι and the Y39A steric gate mutant to incorporate ribonucleotides during DNA synthesis, we initially compared primer extension in the presence of all four dNTPs or NTPs. Because wild-type pol ι is most active in the presence of low concentrations of either magnesium or manganese (15), we assayed the ability of both wild-type pol ι and the Y39A variant to incorporate dNTPs/NTPs in the presence of either 500 μm Mg^2+^ or 250 μm Mn^2+^ over time. As previously reported, in the presence of magnesium, wild-type pol ι efficiently extends the primer by several bases in the presence of dNTPs. In contrast, it exhibits reduced, but nevertheless significant, primer extension when NTPs are present (Fig. 2*A*). However, even after extended reactions times, the major product is just one base longer than the primer. In the presence of magnesium, the pol ι Y39A variant exhibits very low levels of incorporation of dNTPs and even lower levels of incorporation of NTPs (Fig. 2*A*). In dramatic contrast, when manganese is the divalent cation in the reaction mixture, pol ι Y39A efficiently and completely extends the primer to a similar extent with dNTPs or NTPs (Fig. 2*B*). Similarly, in the presence of manganese, wild-type pol ι efficiently extends the primer with dNTPs or NTPs, albeit with reduced efficiency compared with pol ι Y39A (Fig. 2*B*).

##### Fidelity of Ribonucleotide Incorporation Promoted by Wild-type pol ι and the Y39A Steric Gate Mutant

**FIGURE 3.**
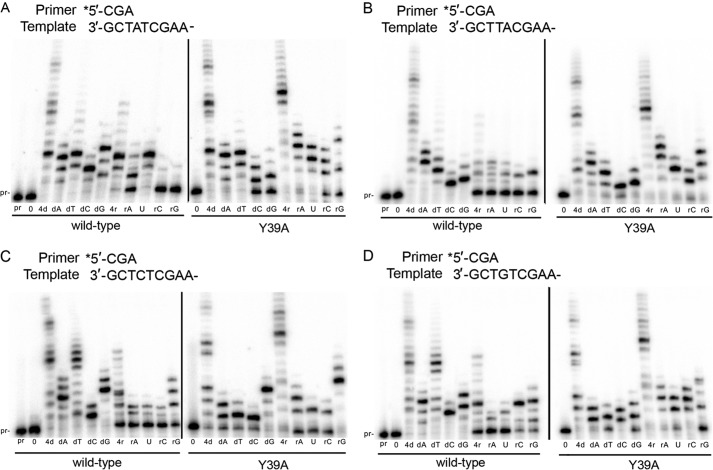
**Removal of steric gate residue results in increased NTP incorporation and higher base selectivity when incorporating individual dNTPs.** *A–D*, incorporation of individual nucleotides by wild-type pol ι or pol ι Y39A on template A (*A*), template T (*B*), template C (*C*), and template G (*D*).

Given that the pol ι Y39A variant exhibits little activity in the presence of magnesium, all subsequent *in vitro* replication reactions were performed in the presence of 250 μm Mn^2+^. To assess the fidelity of wild-type pol ι and the Y39A variant to incorporate dNTPs/NTPs during polymerization, we evaluated extension of a primer in the presence of individual or four NTPs, in parallel with individual or four dNTPs. Wild-type pol ι has been shown to be extremely error-prone when incorporating dNTPs on an undamaged DNA template in the presence of magnesium (5). We observed a similar lack of dNTP selectivity for wild-type pol ι in primer extension assays on all four template bases in the presence of manganese (Fig. 3). Extension by at least one nucleotide is observed for each individual dNTP at each template base, consistent with previous reports (15, 16). In many cases, primers are extended by several nucleotides, indicating multiple misincorporations and subsequent extension of the mispaired bases by the error-prone polymerase. The pattern of NTPs incorporated by wild-type pol ι is clearly different from that observed with dNTPs (Fig. 3). The selectivity for misincorporated NTPs and primer extension lengths are much lower than observed with dNTPs. Our results therefore indicate that although wild-type pol ι has very poor selectivity at the base level for dNTPs, it generally exhibits better selectivity at the base level for NTPs. Incorporation of NTPs by wild-type pol ι appears to be template-dependent, because full primer extension is observed with at least one single NTP on templates A and G, whereas much less NTP incorporation is observed on templates T and C. These results indicate that undamaged DNA templates containing A or G sequences may be at greater risk *in vivo* for NTP incorporation (and the resulting mutagenic or destabilizing consequences) by wild-type pol ι.

Under the same assay conditions, we found that pol ι Y39A incorporates individual NTPs to a much greater extent than wild-type pol ι (Fig. 3). When provided all four NTPs, Y39A synthesizes the primer with a similar efficiency to reactions with all four dNTPs. Regarding incorporation of individual NTPs, Y39A displays considerably reduced base selectivity compared with the wild-type enzyme on all templates. In contrast, however, when incorporating individual dNTPs, Y39A appears to be more selective than wild-type pol ι because extension products in the presence of individual dNTPs are shorter than observed with the wild-type enzyme (Fig. 3). Thus, the pol ι Y39A enzyme exhibits reduced overall discrimination for sugar selectivity but an increased discrimination for the dNTP base.

To facilitate quantitative comparison of nucleotide discrimination differences observed between wild-type pol ι and pol ι Y39A, we performed steady-state kinetic analysis for insertion opposite templates T and G with select nucleotides. As indicated by the primer extension assays, pol ι Y39A exhibits increased discrimination between correct and incorrect dNTPs on both templates (Table 1). At template G, pol ι Y39A incorporates dGTP 13-fold less frequently than the wild-type enzyme. In contrast, our kinetic data show that pol ι Y39A is incapable of distinguishing between dCTP and rCTP when incorporating opposite G, with an *f*_inc_ value of 1 for rCTP. This lack of discrimination makes pol ι Y39A 1100-fold more efficient than wild-type pol ι for rCTP misincorporation. However, pol ι Y39A is capable of discriminating between rCTP and rGTP, and misincorporates rGTP opposite template G only 10-fold more efficiently than wild-type pol ι (Table 1). Removal of the steric gate constraint in the pol ι Y39A enzyme not only makes it a more accurate DNA polymerase but also confers the ability to efficiently incorporate correctly matched NTPs.

**TABLE 1:** **Steady-state kinetics of ribonucleotide incorporation opposite templates G and T** *k*_cat_, *K_m_*, and *k*_cat_/*K_m_* values are reported as the averages of three experiments ± the standard error of the mean.

Nucleotide	Enzyme	*k*_cat_	*K_m_*	*k*_cat_/*K_m_*	*f*_ind_*^a^*	Fold change
		*min*^−*1*^	μ*m*			
Template G						
dCTP	WT	24 ± 3	0.037 ± 0.003	670 ± 60		
	Y39A	9.0 ± 2.1	0.23 ± 0.03	37 ± 10		
dGTP	WT	8.0 ± 0.7	0.83 ± 0.17	10 ± 2	0.016	(13)*^b^*
	Y39A	0.22 ± 0.01	5.0 ± 0.6	0.050 ± 0.010	0.0012	
rCTP	WT	0.077 ± 0.003	0.14 ± 0.03	0.60 ± 0.15	0.00090	1100
	Y39A	4.7 ± 1.2	0.13 ± 0.04	38 ± 6	1.0	
rGTP	WT	0.070 ± 0.010	5.1 ± 0.5	0.014 ± 0.001	0.000021	10
	Y39A	0.043 ± 0.007	5.3 ± 0.3	0.0081 ± 0.0012	0.00021	
Template T						
dATP	WT	24 ± 3	0.037 ± 0.007	670 ± 40		
	Y39A	6.7 ± 0.2	0.97 ± 0.15	7.2 ± 1.1		
dGTP	WT	9.0 ± 0.3	0.0083 ± 0.0017	1200 ± 300	1.8	(5)
	Y39A	2.3 ± 0.4	0.87 ± 0.13	2.9 ± 0.8	0.40	
rATP	WT	ND*^c^*	ND			>1885*^d^*
	Y39A	14 ± 3	4.0 ± 0.6	3.6 ± 0.8	0.49	
rGTP	WT	0.67 ± 0.09	4.3 ± 1.2	0.18 ± 0.04	0.00026	850
	Y39A	6.5 ± 1.0	4.0 ± 0.6	1.6 ± 0.1	0.22	

*^a^* The nucleotide misincorporation ratio, *f*_inc_ = (*k*_cat_/*K_m_*)_incorrect_/(*k*_cat_/*K_m_*)_correct_.

*^b^* Fold changes within parentheses indicate a greater misincorporation propensity by WT compared to Y39A.

*^c^* ND, not detectable (indistinguishable from control assays in the absence of nucleotides).

*^d^* rATP incorporation for wild-type pol ι was calculated by taking the lowest observed *f*_inc_ value on template T and using that as the wild-type value, so fold change relative to Y39A is greater than 1885-fold.

With regards to template T, wild-type pol ι exhibits its hallmark preferential dGTP incorporation (by nearly 2-fold relative to dATP), whereas pol ι Y39A has lost this unique signature. Indeed, pol ι Y39A has reduced misincorporation propensity compared with the wild-type enzyme for dG:T misincorporation, as indicated by a 5-fold lower *f*_inc_ value. When presented with NTPs, pol ι Y39A incorporates rGTP 850-fold more efficiently than wild-type pol ι. The base preference of wild-type pol ι for incorporation of G opposite template T is retained, however, because incorporation was observed at low levels for rGTP but was undetectable for rATP. In contrast, pol ι Y39A inserts rATP more efficiently than rGTP (Table 1).

##### Wild-type pol ι Is Able to Extend a Ribonucleotide-terminated Primer

**FIGURE 4.**
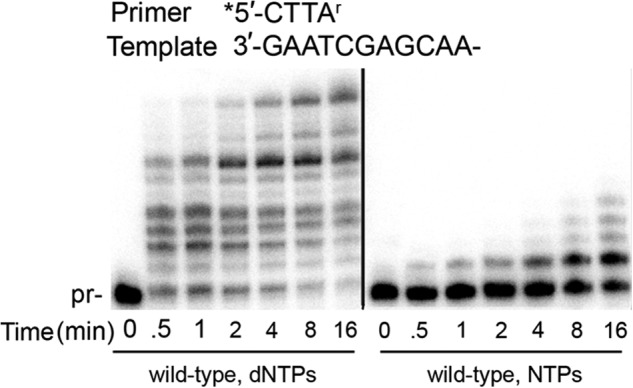
**Wild-type pol ι can efficiently extend a ribonucleotide-terminated primer with dNTPs.** When provided NTPs, wild-type exhibits reduced extension. Primer extension assays are shown using a DNA substrate with a rATP-terminated primer (A^r^) and the indicated nucleotides.

NTP incorporation was observed with both wild-type pol ι and pol ι Y39A enzymes, but less extension was observed when wild-type pol ι was only provided with NTPs. Therefore, we investigated the ability of wild-type pol ι to extend a primer with a terminal ribonucleotide (rATP) correctly base-paired with a template T. In these modified primer extension reactions, we sought to determine whether wild-type pol ι would terminate after NTP incorporation or whether NTPs would be extended with either dNTPs or NTPs and thus persist in the replicated DNA. The reactions were performed with either all four dNTPs or all four NTPs under the same conditions as described above over a 15-min time course. Wild-type pol ι efficiently extends a ribonucleotide-terminated primer when provided dNTPs, with nearly all primer utilized by 30 s (Fig. 4). In contrast, only a small amount of extension through NTP incorporation by wild-type pol ι is observed in this context. These results suggest that a ribonucleotide inserted by wild-type pol ι *in vivo* may persist in DNA after being efficiently extended by further dNTP incorporation.

##### Wild-type pol ι Is Capable of Ribonucleotide Incorporation Opposite Damaged DNA

**FIGURE 5.**
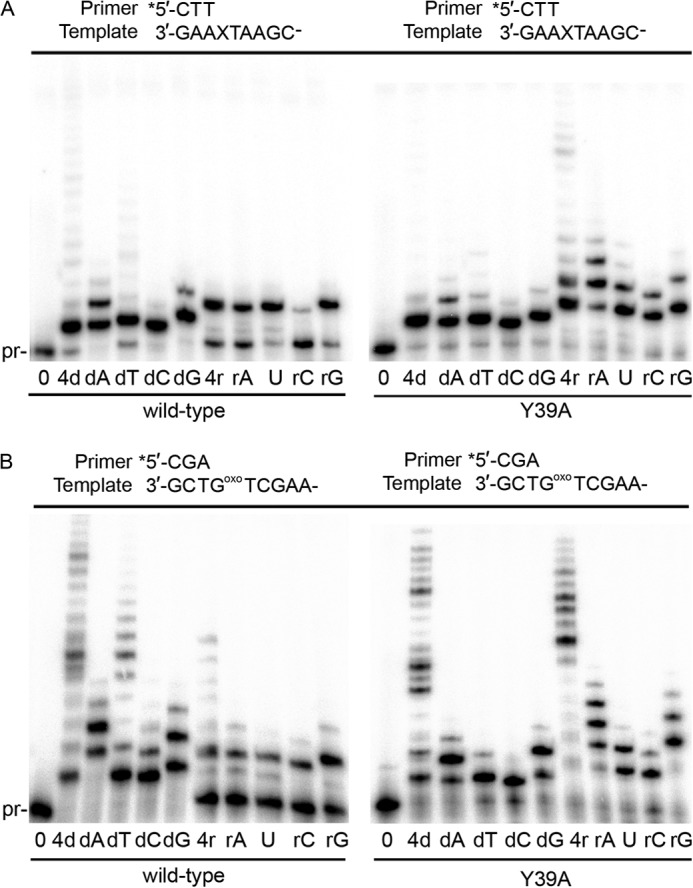
**pol ι can incorporate NTPs opposite damaged DNA.** *A*, wild-type pol ι and pol ι Y39A incorporate NTPs opposite an abasic site template. *B*, wild-type pol ι and pol ι Y39A incorporate NTPs opposite an 8oxoG template.

**FIGURE 6.**
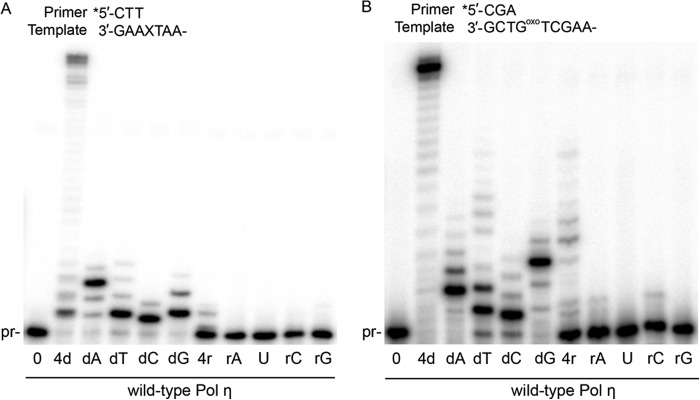
**Wild-type pol η does not incorporate NTPs opposite an abasic site (*A*) or 8oxoG template (*B*).**

To assess the ability of wild-type pol ι and pol ι Y39A to incorporate NTPs during TLS, we performed primer extension assays using DNA substrates Temp8oxoG and XTA48. We observe marked incorporation of individual NTPs by wild-type pol ι opposite both abasic site (Fig. 5*A*) and 8oxoG (Fig. 5*B*) templates. Interestingly, wild-type pol ι will readily incorporate rATP, UTP, and rGTP opposite an abasic site, but it does not incorporate rCTP. Without an effective steric gate, the pol ι Y39A variant readily incorporates all four NTPs opposite the abasic site. Incorporation of NTPs by wild-type pol ι is also observed on the 8oxoG template and, again, to a greater extent with Y39A. To put these remarkable observations into perspective, the related Y family TLS polymerase pol η only incorporates miniscule levels of NTPs opposite either an abasic site or an 8oxoG lesion (Fig. 6), indicating that the ability of wild-type pol ι to efficiently incorporate ribonucleotides during lesion bypass is not common among TLS polymerases.

##### The pol ι Y39A Substitution Does Not Affect DNA Binding Affinity in Vitro or Recruitment to Replication Foci in Vivo

**FIGURE 7.**
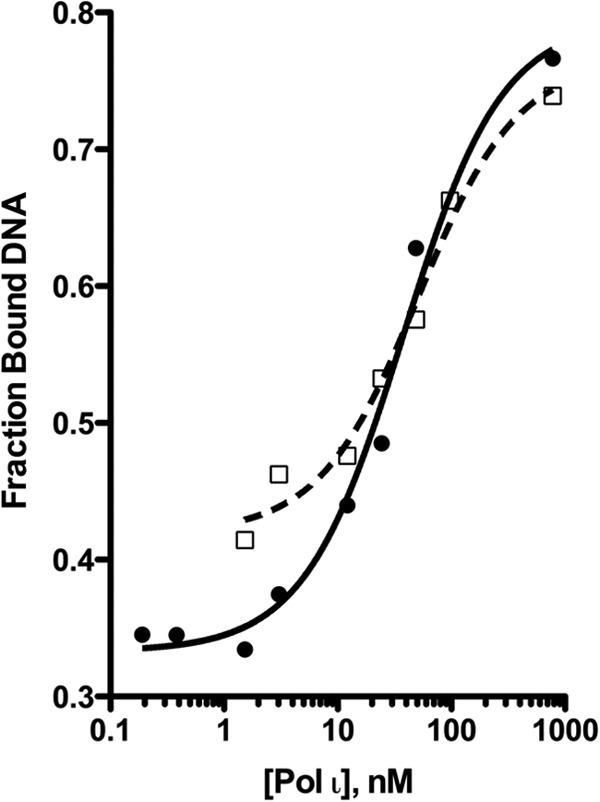
**pol ι Y39A has a similar binding affinity for DNA as wild-type pol ι.** Varying enzyme concentrations were incubated with 0.2 nm DNA for 20 min at 25 °C. The data were plotted as fractions of bound DNA as a function of enzyme concentration to obtain the dissociation constant *K_D_*_(DNA)_. Wild-type pol ι is indicated by the *solid line* and ●, and pol ι Y39A is indicated by the *dashed line* and □. The *K_D_*_(DNA)_ values for wild-type pol ι and pol ι Y39A are 38 ± 7 and 51 ± 12 nm, respectively.

To determine the effect of the single amino acid Y39A substitution on the DNA binding capacity of pol ι, we determined the DNA binding affinity constants for wild-type pol ι and Y39A pol ι using a gel mobility shift assay (Fig. 7). In this assay, wild-type pol ι has a *K_D_*_(DNA)_ of 38 ± 7 nm, which is in accordance with previously reported results obtained using active site titration assays (36). We observed a *K_D_*_(DNA)_ of 51 ± 12 nm for pol ι Y39A; therefore the pol ι Y39A enzyme binds DNA with similar affinity as the wild-type enzyme. These results indicate that the aberrant incorporation activity of pol ι Y39A is not a result of altered DNA binding affinity. The Y39A substitution, although disruptive to nucleotide discrimination, does not affect the ability of pol ι to bind DNA.

**FIGURE 8.**
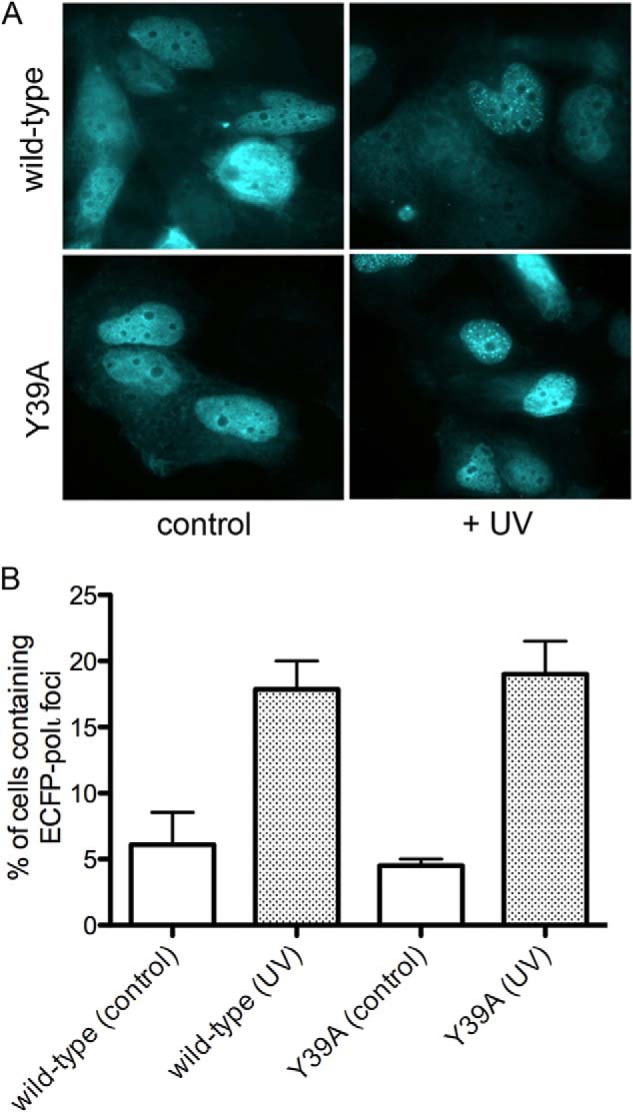
**pol ι Y39A is recruited to replication foci following UV treatment similar to wild-type pol ι.** *A*, upon UV treatment, both wild-type pol ι and pol ι Y39A-expressing cells exhibit a significant increase in nuclear replication foci. *B*, no significant difference is observed between wild-type pol ι and pol ι Y39A under either condition.

To test the hypothesis that the Y39A substitution does not disrupt global structure or cellular properties of the enzyme, we evaluated the recruitment of pol ι Y39A to damage-induced nuclear replication foci. Wild-type pol ι has been previously shown to form discrete nuclear foci in response to UV exposure, and this recruitment is likely due to direct interaction with DNA pol η (33). MRC5 cells were transfected with a CFP-pol ι fusion protein coding for either the wild type or Y39A substitution. Cells were subsequently irradiated with 7 J/m^2^ UV and allowed to recover for 6 h before evaluating for the presence of nuclear CFP foci. As expected, cells expressing the wild-type fusion protein exhibit a significant increase in foci upon UV treatment (Fig. 8). A similar increase in foci was observed in cells expressing the CFP-pol ι Y39A fusion protein. No significant difference was observed across the treated or untreated groups, indicating that pol ι Y39A exhibits a cellular response to DNA damage similar to wild-type pol ι. Therefore the Y39A substitution does not affect the recruitment of the enzyme to damage-induced foci, indicating that the enzyme is not grossly misfolded and retains appropriate protein-protein interactions *in vivo*.

## DISCUSSION

Incorporation of ribonucleotides into DNA has mutagenic consequences, because ribonucleotides are unstable and make the DNA susceptible to strand breaks. Replicative polymerases have evolved active site configurations that efficiently exclude the incorporation of NTPs. This active site configuration typically includes an aromatic residue that functions as a steric gate, a physical barrier that provides a steric clash with the 2′-OH group that distinguishes NTPs from dNTPs. The steric gate residue has been implicated to have other specialized functions that vary depending on the individual polymerase (37). Depending on the identity and primary function of the polymerase, the steric gate residue may be more or less efficient at excluding NTPs from being incorporated during polymerization. For example, wild-type *E. coli* pol V can incorporate rNTPs without compromising efficiency, resulting in synthesis of long RNA tracts (25).

In this study, we investigated the mechanism of sugar discrimination employed by the human Y family TLS polymerase, pol ι. We found that wild-type pol ι is capable of incorporating NTPs opposite undamaged DNA depending on the sequence context. pol ι also readily incorporates NTPs opposite damaged DNA, including an abasic site and an 8oxoG lesion. Replicative polymerases generally adhere to the “A rule” by preferentially incorporating dA opposite an abasic site (38–41). However, pol ι has been shown to exhibit unique behavior when replicating across an abasic site in the presence of dNTPs (42, 43). pol ι does not follow the A rule, because kinetic data show a preference for pol ι to insert dG, followed by dT, dA, and dC, and structural data indicate that pol ι can stabilize the incoming dNTP in the absence of a templating base. Our results with individual NTP incorporation were similar; when incorporating NTPs opposite an abasic site, we found that pol ι has a preference for rG and U. pol ι is less efficient at incorporating rA opposite an abasic site, and almost no incorporation of rC is observed. No extension was observed beyond the base incorporated opposite the basic site, consistent with previous dNTP studies (43). Given that *in vivo*, pol ι will bypass a lesion and continue to synthesize a short stretch of nucleotides beyond the lesion, our results indicate that certain DNA sequence contexts may be more “at risk” for ribonucleotide incorporation—both at the site of damage itself and the undamaged downstream nucleotides. The ability of wild-type pol ι to incorporate NTPs has significant *in vivo* considerations, given that the cellular nucleotide pool is highly biased; roughly 10–100-fold more NTPs are present compared with the corresponding dNTP (17, 18).

To further investigate the structural basis for NTP discrimination, we mutated the putative steric gate residue Tyr^39^ of pol ι to Ala. This alteration effectively removes the steric clash that would result between the 2′-OH of the NTP and the aromatic steric gate residue. Removal of the steric gate makes the Y39A enzyme much more efficient than wild-type pol ι at incorporating and extending NTPs opposite both undamaged and damaged DNA. Surprisingly, in replication assays pol ι Y39A has reduced sugar selectivity but exhibits increased base selectivity when incorporating dNTPs. Steady-state kinetic analysis confirmed that pol ι Y39A cannot discern a difference between the dNTP and NTP version of the same base. Moreover, pol ι Y39A has a stronger preference for traditional Watson-Crick base pairing. When incorporating opposite template T, wild-type pol ι exhibits a classical 2-fold increase in incorporation efficiency for the T:dG mispair. In contrast, pol ι Y39A is more efficient at incorporating dA opposite template T, making it less mutagenic than the wild-type enzyme.

**FIGURE 9.**
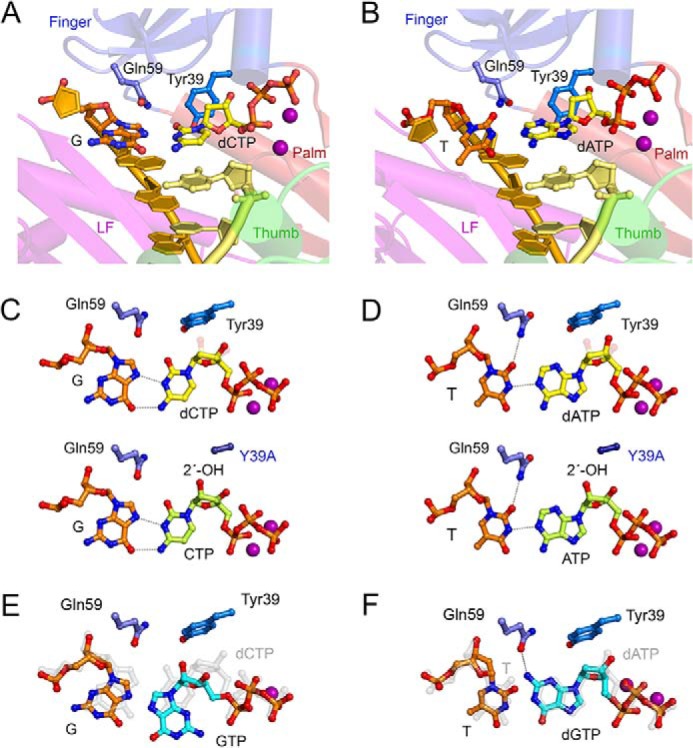
*A*, pol ι ternary complex with template dG and incoming dCTP (PDB code 2ALZ). pol ι is shown in ribbon diagram with the four structural domains palm, finger, thumb, and little finger (*LF*) color-coded. The DNA template is shown in *orange*, and primer is in *yellow*. The replicating base pair and the side chains of Gln^59^ and Tyr^39^ are shown in multicolor *ball-and-stick models*, where *red* represents oxygen atoms, and *blue* represents nitrogen. The Mg^2+^ ions in the active site are shown as *purple spheres. B*, pol ι-template dT and incoming dATP shown in the same scheme as in *A*. The structure is modeled after the BrU/dGTP structure (PDB code 3H4D). *C* and *D*, the zoomed in views (approximately orthogonal to *A* and *B*) of the replicating base pairs in the pol ι complexes. *E*, the G:dGTP mismatch removes interaction of the steric gate of pol ι and the incoming nucleotide. *F*, the T:dGTP mismatch is accommodated and even preferred by pol ι.

Unlike other Y family polymerases, which have a spacious active site, the constricted active site of pol ι is biased against normal Watson-Crick base pairing. When inserting a dCTP opposite a template G, as shown in Fig. 9*A*, the template adopts the *syn* conformation because the narrow active site is too small for a normal Watson-Crick base pair. When the nascent base pair is T:dATP (Fig. 9*B*), the template T is tilted and nonplanar relative to dATP because of the narrow active site. Orthogonal views of template G (Fig. 9*C*) and template T (Fig. 9*D*) highlight the steric gate effect of residue Tyr^39^ that is removed when the residue is changed to the much smaller residue, Ala (Y39A). Fig. 9 (*A* and *B*) highlights the atypical hydrogen bonds between the G:dCTP and T:dATP base pairs, as well as the hydrogen bond between Gln^59^ and the template dT, which may stabilize the tilted template dT. If present, the 2′-OH of ribonucleotides CTP or ATP (shown in *green* carbon and *red* oxygen) would clash badly with Tyr^39^ in the wild-type enzyme. Fig. 9 (*C* and *D*) shows that CTP and ATP can be accommodated in the pol ι Y39A mutant, because the steric clash with the 2′-OH has been eliminated.

In the G:dGTP mispair case (Fig. 9*E*), both template and incoming dGTP are likely to shift from the G:dCTP position (shown in semitransparent *gray*). The shifts of both alleviate the steric clashes between Tyr^39^ and the incoming nucleotide and thereby reduce the base selection, as well as discrimination against an incoming ribonucleotide by the wild-type pol ι enzyme. The shifts and reduced interactions with the enzyme via Tyr^39^ may also explain the much reduced *k*_cat_ and increased *K_m_* of both wild-type pol ι and pol ι Y39A when misincorporating GTP opposite a template G, because neither enzyme efficiently performs this misincorporations (Table 1). The T:dGTP mismatch, however, is readily accommodated, because the structure of the active site of pol ι favors mispairing (Fig. 9*F*). In this case, dGTP forms a hydrogen bond with Gln^59^. For comparison, the normal T:dATP base pair is shown in semitransparent *gray overlay*. When incorporating dNTPs, base selectivity is increased with Y39A because of the increased size of the active site that can properly accommodate and thus favor an incoming nucleotide, in the correct Watson-Crick base pairing configuration. Gln^59^ may hydrogen bond with the template base (T) rather than with the incoming nucleotide (dGTP) (Fig. 9, *D*, *bottom panel*, and *F*). The Y39A substitution may also favor Mn^2+^ over Mg^2+^ because the relaxed coordination geometry of Mn^2+^ is able to stabilize a comparatively less constricted active site. The narrower and more constrained active site of wild-type pol ι coordinated with Mg^2+^ provides incoming nucleotide positioning that allows for polymerization but concomitantly favors mutagenic base pairing.

Therefore, pol ι Y39A exhibits a seemingly mixed phenotype, reduced sugar discrimination with increased base discrimination. Previous studies on *E. coli* pol V have shown that the same two phenotypes can be attributed to two different residues (25). The pol V residue that corresponds to Tyr^39^ of pol ι is pol V Y11. The pol V Y11A mutant has been shown to have a marked reduction in sugar discrimination relative to the wild-type enzyme, but its base selectivity remained unchanged. In contrast, alteration of the neighboring residue, Phe^10^ to Leu, had the effect of increasing both base and sugar selectivity for pol V. Our studies with pol ι suggest that amino acid substitutions at the steric gate of a DNA polymerase can have pronounced effects on both sugar and base selectivity, as well as metal ion coordination.

During DNA replication in yeast, replicative polymerases have been estimated to incorporate ribonucleotides at a rate of 1 per 625–5,000 bases replicated, leading to over 10,000 ribonucleotides incorporated into the genome during a single round of replication (20). Ribonucleotide incorporation may also occur during DNA repair. DNA polymerase β, the main polymerase involved in base excision repair, has been estimated to incorporate NTPs at a rate of one per 81 insertion events. Ribonucleotide incorporation by pol ι during TLS may further contribute to this burden. Mechanisms of NMP removal from DNA have recently been described in yeast and mice and are viewed as essential to maintaining genome integrity (19, 23). Although the presence of NMPs in DNA is mutagenic and may also contribute to genomic instability, it has also been recently shown to act as a signal for DNA mismatch repair (35, 44). Therefore, a physiological balance may exist between incorporation and excision of ribonucleotides from the genome. Although NTP incorporation during TLS synthesis by pol ι may contribute to the lesion burden, we acknowledge the intriguing possibility of ribonucleotide incorporation by pol ι as a signal for further repair.

## Footnotes

2 The abbreviations used are:

pol: polymerase
TLS: translesion DNA synthesis
PDB: Protein Data Bank.
